# A reference interval for serum IgG subclasses in Chinese children

**DOI:** 10.1371/journal.pone.0192923

**Published:** 2018-03-05

**Authors:** Zhongjuan Liu, Chuiwen Deng, Ping Li, Jingxia Wang, Lijuan Ma, Yongzhe Li, Yingchun Xu

**Affiliations:** 1 Department of Clinical Laboratory, Peking Union Medical College Hospital, Chinese Academy of Medical Sciences & Peking Union Medical College, Beijing, China; 2 Department of Rheumatology and Clinical Immunology, Peking Union Medical College Hospital, Chinese Academy of Medical Sciences & Peking Union Medical College, Beijing, China; 3 Key Laboratory of Rheumatology and Clinical Immunology, Ministry of Education, Beijing, China; 4 Department of Clinical Laboratory, Capital Institute of Pediatrics, Beijing, China; Laboratoire d’Immunologie CHU Rangueil et Université Toulouse III Toulouse, FRANCE

## Abstract

**Background:**

Reference intervals (RIs) for serum IgG subclasses vary greatly among different geographical regions. The present study aimed to establish RIs for serum IgG subclasses in Chinese children, which is essential for interpretation of laboratory findings and making clinical decisions.

**Methods:**

This study was performed in accordance with guideline C28-A3, proposed by the International Federation of Clinical Chemistry and the Clinical and Laboratory Standards Institute. In total, 607 apparently healthy Chinese children were enrolled, and serum levels of IgG subclasses were measured. Individuals were stratified by age and the RIs were determined through statistical analysis.

**Results:**

Following were the median values of RIs for serum IgG subclasses in Chinese children: IgG1, 2.78 g/L; IgG2, 0.85 g/L; IgG3, 0.13 g/L; IgG4, 0.06 g/L at 1–6 months of age; IgG1, 3.64 g/L; IgG2, 0.73 g/L; IgG3, 0.19 g/L; IgG4, 0.03 g/L at 6–12 months of age; IgG1, 5.15 g/L; IgG2, 0.87 g/L; IgG3, 0.19 g/L; IgG4, 0.07 g/L at 1–2 years of age; IgG1, 5.26 g/L; IgG2, 1.23 g/L; IgG3, 0.14 g/L; IgG4, 0.11 g/L at 2–3 years of age; IgG1, 6.33 g/L; IgG2, 1.8 g/L; IgG3, 0.2 g/L; IgG4, 0.21 g/L at age 3–4 years; IgG1, 7.05 g/L; IgG2, 1.87 g/L; IgG3, 0.25 g/L; IgG4, 0.29 g/L at 4–6 years of age; IgG1, 6.19 g/L; IgG2, 1.93 g/L; IgG3, 0.2 g/L; IgG4, 0.28 g/L at 6–9 years of age; IgG1, 6.76 g/L; IgG2, 2.29 g/L; IgG3, 0.27 g/L; IgG4, 0.37 g/L at 10–12 years of age; IgG1, 7.45 g/L; IgG2, 2.92 g/L; IgG3, 0.28 g/L; IgG4, 0.38 g/L at 13–16 years of age.

**Conclusion:**

To our knowledge, this study is the first to establish RIs for serum IgG subclasses exclusively in Chinese children.

## Introduction

Fifty years ago, four IgG subclasses were identified on the basis of unique heavy-chain antigenic epitopes. A subsequent study reported differences in serum titers among these antibodies [1]. Since then, IgG subclasses have received increasing attention owing to its association with deficiency disease and IgG4-related disease, especially among children [2].

IgG-subclass deficiency is defined as a deficiency in one or more IgG-subclasses (>2 SD below age- matched reference values) with normal or near normal IgG titers [3]. IgG subclasses play a vital role in not only clinical decision making but also defining the nature of the immunodeficiency disease. For example, detection of IgG subclasses may help determine one’s susceptibility to infections. Children deficient in IgG2 or IgG3 would not display a satisfactory response to vaccines, especially polysaccharide vaccines, compared to those with normal IgG2 or IgG3 titers [4]. In addition, deficiencies are classified into different subtypes on the basis of IgG subclasses, according to the classification committee guidelines of the International Union of Immunological Societies [5]. Serum levels of IgG subclasses are widely considered as diagnostic parameters for IgG4-related disease [6], which occurs typically among elder adults; however, cases of IgG4-related disease have been reported among children, predominantly women [7].

Hence, accurate determination of an RI for the levels of serum IgG subclasses is important for making clinical decisions in immunodeficiency disease and IgG4-related disease. To our knowledge, several related studies have attempted to determine RIs for serum IgG subclasses in children [8–12]. Three of them established RIs through the nephelometric method, and these studies were conducted in Turkey, Canada, and Hong Kong and yielded diverse results, while others used enzyme-linked immunosorbent assays or radial immunodiffusion assays [8–10].

Considering that differences in ethnicity or race could yield disparities in RIs for immunologic indices [13], the present study attempted to establish RIs for serum IgG subclasses in apparently healthy children from the mainland of China.

## Materials and methods

### Selection of apparently healthy children from China

This study was designed and carried out in accordance with guideline C28-A3, proposed by the International Federation of Clinical Chemistry and the Clinical and Laboratory Standards Institute [14].

Apparently healthy children from China were selected from a population, based on the following inclusion criteria: (1) age ≤ 16 years; (2) having nomal records of routine blood examination, C-reaction protein and biochemical marker levels of liver and kidney function. Following were the exclusion criteria: (1) recent infection; (2) immune-related diseases, including but not limit to immunodeficiency, allergy, and autoimmune diseases; (3) family history of autoimmune diseases; (4) recent vaccination or treatment with immunosuppressive and/or anti-inflammatory therapy.

From January 2014 to February 2016, children participated in annual physical examinations and patients of some diseases (strabismus, redundant prepuce, and hernia) who underwent preoperative evaluation at Capital Institute of Pediatrics were screened, of which 607 individuals fulfilled the eligible criteria. Hence, 607 apparently healthy children were recruited in the present study and stratified by age in accordance with a previous study [8]. The Ethics Committee of the Peking Union Medical College Hospital approved this study. Written informed consent was waived owing to the nature of the study design, which utilized serum samples obtained through routine tests. Participant identities were blinded after data collection.

### Sample collection

Serum samples were collected from the leftover samples in clinical laboratory tests. Essentially, the participants were required to fast for 6 h before blood collection. The serum samples were collected and frozen at -80 °C until use.

### Assessment of the serum IgG subclass

Analyte detection and statistical analysis were conducted from July 2014 to March 2016. Serum levels of IgG subclasses were estimated through the nephelometric method, using the BNII Nephelometer Analyzer (Dade Behring GmbH, Marburg, Germany) and commercially available kits (Siemens Healthcare Diagnostics Products GmbH, Germany). IgG subclasses were assayed with N antiserum against human IgG, N AS IgG1 (OQXI [lot no. 090167]), N AS IgG2 (OQXK [lot no. 090274]), N Latex IgG3 (OPAV [lot no. 086169]), and N Latex IgG4 (OPAU [086070]).

The N protein standard SL was used to calibrate the assessment of the serum IgG subclass, based on the Sanquin (Amsterdam, Netherlands) nephelometric standard M1590, which could be traced to the WHO 67/97 reference preparation [15].

Inter-assay coefficients of variation (CV) were calculated from the internal quality control (Level SL-H/L/M). The concentrations for the internal quality control were as follows: Level SL-H (lot no. 084845): IgG1 = 7.33g/L, IgG2 = 3.87 g/L, IgG3 = 0.482 g/L, IgG4 = 0.779 g/L; Level SL-L (lot no. 084644): IgG1 = 3.78 g/L, IgG2 = 1.94 g/L, IgG3 = 0.191 g/L, IgG4 = 0.479 g/L; and Level SL-M (lot no. 084746): IgG1 = 5.83 g/L, IgG2 = 2.85g/L, IgG3 = 0.333 g/L, IgG4 = 0.686g/L.

### Statistical analysis

Descriptive statistics and percentiles were calculated using SPSS 19.0, the two reference limits being the 2.5th and 97.5th percentiles for the reference population.

Serum IgG subclass RIs were calculated in accordance with the following two steps per our previous study [16]. First, a preliminary RI was calculated from all the data. The outliers were eliminated through the Dixon method [17]. Second, the data were stratified by age (1–6 months, 6–12 months, and 1–2, 2–3, 3–4, 4–6, 6–9, 10–12, and 13–16 years); thereafter, RI of each IgG subclass was calculated.

## Results

### Study population

In total, 607 (304 female; 303 male) apparently healthy Chinese children aged 1 month to 16 years were recruited and stratified by age. There were no significant differences in mean age between the male and female groups.

### RIs for the Chinese children

**Fig 1:**
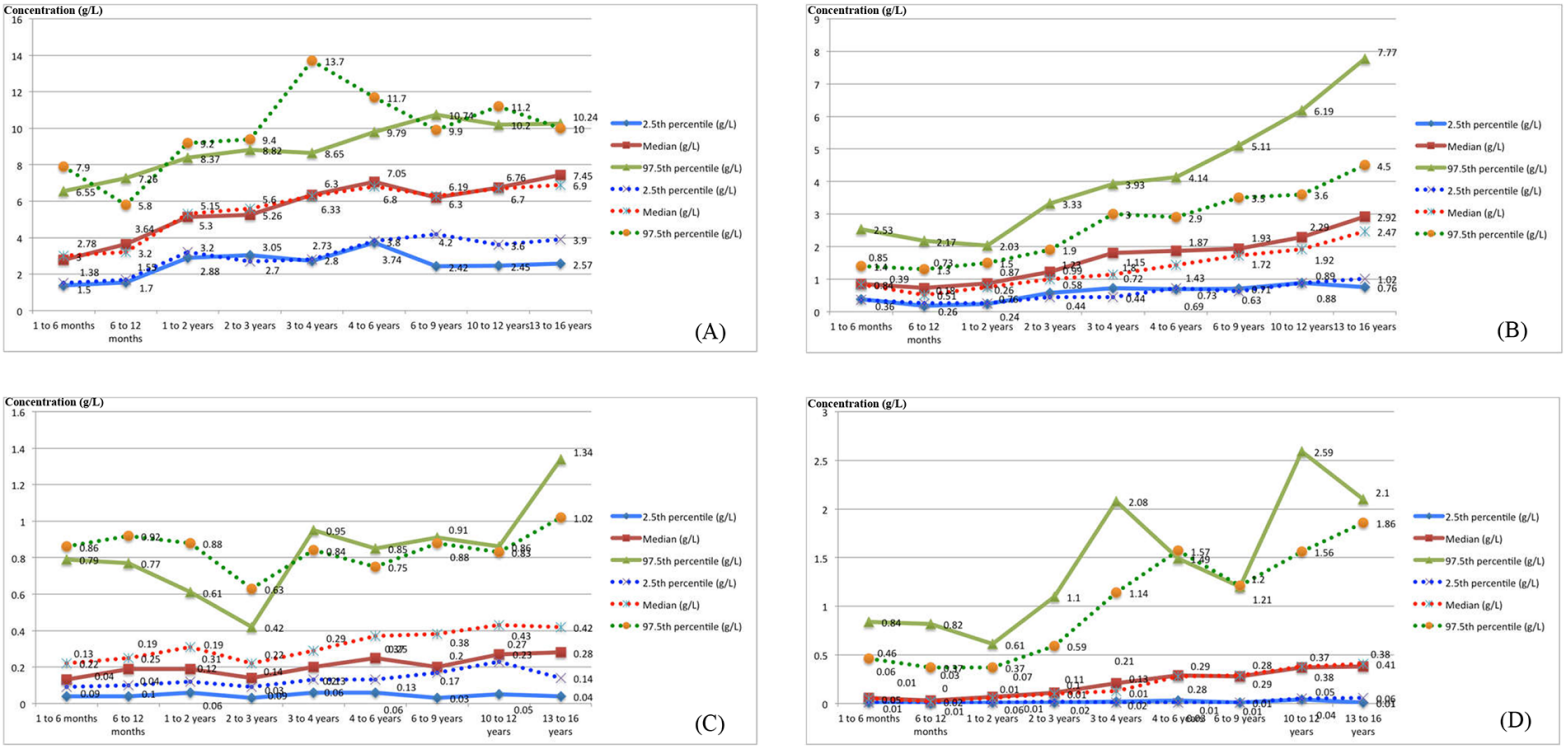
Comparison of data between the present study and a recent study. Comparison of (A) IgG1, (B) IgG2, (C) IgG3, and (D) IgG4 titres. The dotted line indicated the results of Lepage et al.[8], and the solid line the results of the present study.

The age-specific RIs for serum IgG subclasses in the Chinese children are shown in Table 1. No outlier was observed. Our data were compared with those of a recent study that applied the same age stratification (Fig 1) [8]. The inter-assay CVs were 1.9–5.3% with a total CV of 2.6–6.2%.

**Table 1:** Reference interval of serum immunoglobulin G subclass in the Chinese children.

Age group	Number of patients (Male/Female)	2.5th percentile (g/L)	Median (g/L)	97.5th percentile (g/L)
1 to 6 months				
IgG1	31/37	1.38	2.78	6.55
IgG2	31/37	0.39	0.85	2.53
IgG3	31/37	0.04	0.13	0.79
IgG4	31/37	0.01	0.06	0.84
6 to 12 months				
IgG1	25/23	1.55	3.64	7.26
IgG2	25/23	0.18	0.73	2.17
IgG3	25/23	0.04	0.19	0.77
IgG4	25/23	0	0.03	0.82
1 to 2 years				
IgG1	34/33	2.88	5.15	8.37
IgG2	34/33	0.24	0.87	2.03
IgG3	34/33	0.06	0.19	0.61
IgG4	34/33	0.01	0.07	0.61
2 to 3 years				
IgG1	35/34	3.05	5.26	8.82
IgG2	35/34	0.58	1.23	3.33
IgG3	35/34	0.03	0.14	0.42
IgG4	35/34	0.01	0.11	1.1
3 to 4 years				
IgG1	22/26	2.73	6.33	8.65
IgG2	22/26	0.72	1.8	3.93
IgG3	22/26	0.06	0.2	0.95
IgG4	22/26	0.02	0.21	2.08
4 to 6 years				
IgG1	46/37	3.74	7.05	9.79
IgG2	46/37	0.69	1.87	4.14
IgG3	46/37	0.06	0.25	0.85
IgG4	46/37	0.03	0.29	1.49
6 to 9 years				
IgG1	40/47	2.42	6.19	10.74
IgG2	40/47	0.71	1.93	5.11
IgG3	40/47	0.03	0.2	0.91
IgG4	40/47	0.01	0.28	1.2
10 to 12 years				
IgG1	42/35	2.45	6.76	10.2
IgG2	42/35	0.88	2.29	6.19
IgG3	42/35	0.05	0.27	0.86
IgG4	42/35	0.04	0.37	2.59
13 to 16 years				
IgG1	28/32	2.57	7.45	10.24
IgG2	28/32	0.76	2.92	7.77
IgG3	28/32	0.04	0.28	1.34
IgG4	28/32	0.01	0.38	2.1

Siemens recruited 405 healthy children from central Europe and North America and estimated the levels of serum IgG subclasses. They calculated the 2.5th and 97.5th percentiles as the upper and lower limits, respectively, of RIs for each age group. The RIs calculated in the present study were compared with those provided by the manufacturer (Table 2).

**Table 2:** Comparison of the reference values provided by manufacturer and by our research.

Age group	Provided by manufacturer			Provided by our research		
	Patients (n)	2.5th percentile (g/L)	97.5th percentile (g/L)	Patients (n)	2.5th percentile (g/L)	97.5th percentile (g/L)
1 to 12 months						
IgG1	NA	1.510	7.920	116	1.459	7.251
IgG2	NA	0.260	1.360	116	0.360	2.326
IgG3	NA	0.093	0.920	116	0.040	0.700
IgG4	NA	0.004	0.464	115	0.004	0.721
1 to 3 years						
IgG1	NA	2.650	9.380	136	2.975	8.673
IgG2	NA	0.280	2.160	136	0.476	3.129
IgG3	NA	0.087	0.864	136	0.039	0.568
IgG4	NA	0.009	0.742	136	0.009	0.971
3 to 6 years						
IgG1	NA	3.620	12.280	131	3.744	9.787
IgG2	NA	0.570	2.900	131	0.693	4.138
IgG3	NA	0.129	0.789	130	0.058	1.190
IgG4	NA	0.013	1.446	131	0.028	1.488
6 to 12 years						
IgG1	NA	3.770	11.310	164	2.424	10.450
IgG2	NA	0.680	3.880	164	0.721	5.748
IgG3	NA	0.158	0.890	164	0.046	0.855
IgG4	NA	0.012	1.699	164	0.014	1.594
12 to 18 years						
IgG1	NA	3.620	10.270			
IgG2	NA	0.810	4.720			
IgG3	NA	0.138	1.058			
IgG4	NA	0.049	1.985			
12 to 16 years						
IgG1				60	2.570	10.238
IgG2				60	0.756	7.770
IgG3				60	0.043	1.345
IgG4				60	0.017	2.095

## Discussion

Several previous studies have reported RIs for serum IgG subclasses in individuals in Turkey, Canada, and Hong Kong [8–12]; however, none have attempted to establish those of individuals in Mainland China. Hence, the present study aimed to establish RIs of serum IgG subclasses in Chinese children (Table 1).

Generally, RIs are provided in test kits for clinical use, by the manufacturer. These RIs require calibration in accordance with the population of the region where the kits are used. Moreover, previous studies have reported disparate results for RIs of serum IgG subclasses among different populations from an intuitive viewpoint. Hence, a comparison on the present results with those of previous studies or with the cut-off values provided by the manufacturer would help determine whether these differences are regional or racial. Among all studies, Lepage et al. [8] used the same grouping method as that in the present study. Furthermore, one study reported the RIs of serum IgG subclasses in children from Hong Kong [10]. Therefore, we compared our results with those of these two studies.

On comparing data from the present study with that of the two aforementioned studies using the nephelometric method [8–10], two characteristics were observed. First, the serum levels of IgG3 and IgG4 were lower than those of IgG1 and IgG2, which is concurrent with previous findings. In addition, studies on Caucasian populations reported that serum levels of IgG3 were higher than those of IgG4 [8]; this was observed in all age groups except for the age group of 3 to 16 months in the present study (Fig 1). Second, IgG4 has been reported to fluctuate greatly with age, comparing to other subclasses. However, the IgG2, IgG3, and IgG4 levels varied greatly with age in our study, especially compared to the study performed in Hong Kong [10].

Three factors accounted for the differences between the present results and previous results. First is ethnicity and genetic background, which has been previously reported to cause disparities in serum levels of IgG subclasses [12]. The second factor is regional differences. Even in China, RIs of serum IgG subclasses differ between children from mainland China in the present study and Hong Kong [10]. Lastly, the criteria for recruitment of participants in the present study were different in different studies, which might have led to the discrepancy among studies.

Interestingly, obvious reductions in the 97.5th percentile of IgG3 in the 2–3-year age group and the 97.5th percentile of IgG4 of the 6–9-year age group were observed in both the present and previous studies [8]. In addition, obvious reductions in the 97.5th percentile of IgG4 in the 4–6-year age group and the 10–12-year age group were only observed in the present study. Generally, serum levels of IgG and IgG subclasses are considered to vary with age. Hence, the reductions observed exclusively in the present study might have been influenced by the aforementioned three factors. However, no related studies have attempted to illustrate the mechanism underlying the factors contributing to these reductions. Future studies are required to focus on these reductions to elucidate the mechanisms underlying these variations in IgG subclass; this will provide critical information regarding the application of IgG subclasses in the clinical setting.

The RIs provided by the manufacturer are different from those of our study (Table 2). The difference indicates that not only different ethnicities and geographical backgrounds might result in heterogeneity of RIs, but also RIs for children should be established in each region. The RIs established in the present study could help clinicians increase the accuracy of their clinical decisions regarding Chinese children.

Of note, the assessment of IgG subclass was not standardized, which might have influenced the results or may have imposed restrictions on its application. Standardization has been further hampered owing to lack of internationally recognized reference material for determination of IgG subclasses, and major manufacturers of kits for IgG subclasses use two different calibration strategies. European Reference Materials^®^ DA470 (ERM^®^-DA470) [13] are applied in the Binding Site assays, thereby reporting a higher level of IgG3 and a lower level of IgG4 [18, 19] than the Siemens and Sanquin methods that used other calibration strategies through Sanquin nephelometric standard M1590 [15]. Considering that the calibration strategy might induce a bias among different assays for IgG subclasses of different manufacturers, the present results are only applicable to laboratories that use the same instrument and calibration strategy as in the present study.

Total serum IgG levels were determined and compared with the sum of the four IgG subclasses to determine the accuracy of the assays in previous studies [8–10, 13], including our previous study that aimed to establish RIs for IgG subclasses in Chinese adults [20]. In that study, a significant correlation was obtained between the sum of the IgG subclasses and the measured value of total IgG, which supports the accuracy of the assays [20]. Those results are in accordance with the parameters of the Plasma Proteins Calibrator Traceability provided by Siemens, which describes that the sum of IgG subclasses 1–4 corresponds to the total IgG levels based on ERM^®^-DA470. Based on these conditions, and the fact that our study immediately proceeded the previous one using the same instrument [20], this comparison was not made.

Our study has the following limitations of note. First, the eligible participants were not followed-up in a specified period; hence, we could not rule out the possibility that patients might have undergone an incubation period during recruitment. Second, the differences between studies might not only be attributed to the genetic background, ethnicity, and region, but also could have resulted from random variations. Therefore, additional related studies are required to investigate the pathogenesis of IgG-related diseases and to provide more information regarding other factors that may influence children’s RIs of serum IgG subclasses in China.

In conclusion, this is, to our knowledge, the first study to investigate the RI for serum IgG subclasses in Chinese children. Age-specific RIs were established, and their application may help clinicians make accurate clinical decisions for Chinese children.
